# Sheep Infection Trials with ‘Phase-Locked’ Vpma Expression Variants of *Mycoplasma agalactiae*—Towards Elucidating the Role of a Multigene Family Encoding Variable Surface Lipoproteins in Infection and Disease

**DOI:** 10.3390/microorganisms10040815

**Published:** 2022-04-14

**Authors:** Rohini Chopra-Dewasthaly, Andreas Dagn, Christian Lohinger, René Brunthaler, Martina Flöck, Munkhtsetseg Kargl, Shrilakshmi Hegde, Joachim Spergser, Renate Rosengarten

**Affiliations:** 1 Institute of Microbiology, Department of Pathobiology, University of Veterinary Medicine Vienna, Veterinaerplatz 1, A-1210 Vienna, Austria; andi.dagn@gmx.at (A.D.); christian.lohinger@gmx.net (C.L.); munkhtsetseg.kargl@vetmeduni.ac.at (M.K.); shree.hegde@lstmed.ac.uk (S.H.); joachim.spergser@vetmeduni.ac.at (J.S.); renate.rosengarten@vetmeduni.ac.at (R.R.); 2 Institute of Pathology, Department of Pathobiology, University of Veterinary Medicine Vienna, Veterinaerplatz 1, A-1210 Vienna, Austria; rene.brunthaler@vetmeduni.ac.at; 3 Clinic for Ruminants, Department for Farm Animals and Veterinary Public Health, University of Veterinary Medicine Vienna, Veterinaerplatz 1, A-1210 Vienna, Austria; martina.floeck@vetmeduni.ac.at

**Keywords:** antigenic phase variation, *Mycoplasma agalactiae*, contagious agalactia, mycoplasma pathogenicity, immunohistochemistry

## Abstract

The significance of large multigene families causing high-frequency surface variations in mycoplasmas is not well-understood. Previously, VpmaY and VpmaU clonal variants of the Vpma family of lipoproteins of *M. agalactiae* were compared via experimental sheep infections using the two corresponding ‘Phase-Locked Mutants’. However, nothing is known about the infectivity of the remaining four Vpma expression variants VpmaX, VpmaW, VpmaZ and VpmaV as they were never evaluated in vivo. Here, in vivo infection and disease progression of all six Vpma expressers constituting the Vpma family of type strain PG2 were compared using the corresponding *xer1*-disrupted PLMs expressing single well-characterized Vpmas. Each of the six PLMs were separately evaluated using the intramammary sheep infection model along with the control phase-variable wildtype strain PG2. Thorough bacteriological, pathological and clinical examinations were performed, including assessment of milk quality, quantity and somatic cell counts. Altogether, the results indicated that the inability to vary the Vpma expression phase does not hamper the initiation of infection leading to mastitis for all six PLMs, except for PLMU, which showed a defect in host colonization and multiplication for the first 24 h p.i. and pathological/bacteriological analysis indicated a higher potential for systemic spread for PLMV and PLMX. This is the first study in which all isogenic expression variants of a large mycoplasma multigene family are tested in the natural host.

## 1. Introduction

Mycoplasmas are widespread in nature and can cause serious chronic diseases in humans, animals, and plants. They are minimalist prokaryotes possessing small genomes with limited coding capacity, yet they can cause considerable damage to the livestock industry [1]. Although vaccines are available for some avian and porcine mycoplasma species, successful vaccines have so far been evasive for most of other species, including ruminant pathogens [2,3,4,5]. One of the major factors hampering vaccine development is the frequent variation in mycoplasma surface epitopes that are known to undergo non-stochastic progressive changes during infection and are often implicated in host immune evasion [6,7,8,9]. Despite the fact that the sophisticated molecular mechanisms governing these high-frequency phase variations have been well-characterized in several mycoplasma species, their precise regulation and exact functions during infection and disease progression remains largely unclear [6,10,11].

This study concentrates on the surface antigenic variations in *Mycoplasma agalactiae*, a worldwide significant pathogen of goats and sheep causing mainly mastitis, arthritis and keratoconjunctivitis [12]. Despite being notifiable to OIE (World Organization for Animal Health), efficacious therapeutics and vaccines are evasive, mainly due to the limited knowledge about its pathogenicity mechanisms [4]. However, phase variations caused by the family of Vpma (variable proteins of *Mycoplasma agalactiae*) surface lipoproteins play important roles in the survival and persistence of the pathogen inside the host [10,13]. Site-specific recombination of the *vpma* genes caused by the Xer1 recombinase encoded on the same gene locus, leads to high-frequency switching of the six immunodominant proteins, namely VpmaU, VpmaY, VpmaX, VpmaW, VpmaZ and VpmaV found in the type strain PG2 [14,15,16]. Although Vpma profiles were found to be different in few field strains, for instance strain 5632 possesses 23 *vpma* genes distributed on two separate genomic loci, majority of *M. agalactiae* strains exhibited *vpma* gene repertoires similar to PG2 [17,18]. In order to understand the significance of Vpma switching and the role of Vpma proteins in *M. agalactiae* pathogenicity, ‘phase-locked mutants’ (PLMs) expressing stable single Vpma phenotypes were generated by disrupting the *xer1* recombinase gene of PG2 [15]. 

Previous in vitro studies clearly demonstrated the Vpmas to be the immune evasion proteins of *M. agalactiae,* as it clearly escaped the inhibitory effects of anti-Vpma antibodies by Xer1-mediated high-frequency switching to alternative Vpma proteins. Even in Xer1-disrupted PLMs, the presence of Vpma-specific antibodies led to repression of the target Vpma and induction of new Vpma phenotypes by complex *vpma* rearrangements [8]. Additionally, using in vitro adhesion assays, Vpmas were demonstrated to be the major adhesins of *Mycoplasma agalactiae* mediating differential cell adhesion and invasion of Vpma expression variants [19]. The latter results correlated very well with the outcome of an in vivo infection study where sheep were inoculated with an equal mixture of PLMU and PLMY, expressing VpmaY and VpmaU, respectively. These experimental infections underscored the importance of Vpma switching for survival and persistence inside the immunocompetent host and demonstrated the differential infection potential of Vpma variants [10,13]. However, the other four Vpma expression variants were never checked in vivo, and nothing is known about their infectivity or role in disease progression.

In the present study, the infection profile of all six Vpma expressers found in the pathogenic strain PG2, expressing either VpmaU, VpmaV, VpmaW, VpmaX, VpmaY or VpmaZ, was compared in vivo. This was carried out to understand the function of individual Vpma lipoproteins in the natural host during real infection processes, including colonization, tissue tropism, immune evasion and severity of symptoms and disease progression. For this, each of the six corresponding *xer1*-disrupted PLMs were separately evaluated in experimental intramammary sheep infections, parallel to a positive control infection group inoculated with the wildtype strain PG2 that is capable of high-frequency Vpma variations due to the presence of an intact *xer1* gene. Altogether, the results demonstrated that (i) individual PLMs are able to initiate an infection, (ii) all PLM infections caused mastitis with changes in milk quality and quantity visible within one day post infectionem (p.i.), (iii) all PLMs were comparable to the wildtype strain PG2 recovered in high concentrations from milk samples until the day of necropsy, except for PLMU which showed an initial rapid decline in mycoplasma counts in the first 24 h p.i., (iv) PLMV showed a maximum number of positive re-isolations from the necropsied organs and lymph nodes (LNs), and (v) PLMV and PLMX showed significant pathological alterations compared to other groups, indicative of better spread and higher pathogenicity. These two were the only PLMs for which the infection had spread to the left udder halves, as indicated by excretion in milk, and also by their isolation from the left supramammary LN and/or left udders.

## 2. Materials and Methods

### 2.1. Mycoplasma Strains, Animals and Experimental Infection

*M. agalactiae* type strain PG2 and each of the six PLMs, namely PLMU, PLMX, PLMY, PLMW, PLMV and PLMZ, were used for separate infection of groups of three merino sheep, each via the right teat canal with 10^9^ CFU/sheep in 5 mL PBS (Gibco, Life Technologies, Vienna, Austria), as described before [10,19]. All sheep were bred at the VetFarm (former: Teaching and Research Farm Kremesberg) of the University of Veterinary Medicine Vienna and were in the first lactation phase. A group of three sheep infected with PBS served as the negative control. All sheep groups were housed in separate compartments of a biosafety level (BSL) 2 facility one week before inoculation, and infections were carried out in accordance with the guidelines of the institutional ethics committee of the University of Veterinary Medicine Vienna. The whole experiment was carried out in two parts, each part consisting of three randomly chosen PLM-infected groups accompanied by a positive control group infected with wild type PG2 and one negative group inoculated with PBS (Table 1). Part 1 (A) was carried out in September on Merino meat sheep, whereas Part 2 (B) was performed on Merino land sheep in April of the following year. The animals were negative for major sheep bacterial pathogens (confirmed by routine bacteriological diagnostic analysis) and were also confirmed to be seronegative for *M. agalactiae* by ELISA (Cypress Diagnostics, Langdorp, Belgium; IDEXX, Montpellier, France). Sheep were also subjected to regular clinical and serological examinations one week prior to infection and during the entire experimental period of 2 weeks. In addition, prior to the intramammary route infection, the possibility of an existing subclinical mastitis was excluded in the lactating ewes by checking their milk via general bacteriological examination and for somatic cell counts (FOSS ELECTRIC, Hillerôd, Denmark). Prior to infection all animals were dewormed (Ivermectin 0.2 mg/Kg body weight, Ivomec^®^, Boehringer, Ingelheim, Germany). Excretion of bacteria in milk was determined regularly by quantitative bacteriological examination, and colonization of the eye, ear and nose was checked qualitatively and quantitatively from the respective swabs. Colonies were randomly picked from agar plates at regular intervals and confirmed by *M. agalactiae* species specific PCR [20]. All infected animals were euthanized and necropsied between Day 15 and Day 18 p.i. to quantify *M. agalactiae* in various LNs and tissues/organs.

### 2.2. Ethic Statement

The animal experiment was approved by the Ethics Committee of the University of Veterinary Medicine Vienna (§12 of Law for Animal Experiments) and the former Austrian Federal Ministry for Science, Research and Economy (BMWFW-68.205/0106-WF/II/3b/2014). 

### 2.3. Clinical Examination

Table 2 provides an overview of following the clinical course of infection and sample collection. Samples (milk, blood and conjunctival, ear, nasal and genital swabs) were collected regularly, and rectal temperatures measured every day starting from Day 5 before inoculation until the end of the experiment. Clinical examinations including general behavior, internal body temperature, pulse, breathing, ingestion and udder and milk assessments were performed and recorded every day after infection, as per clinical propaedeutics. To quantify clinical signs, a scoring system for general behavior and well-being (Table 3), milk consistency (Table 4) and the clinical status of the udder (4-normal udder; 0-severe mastitis) (Table 5) was implemented, as described ahead. Scores were calculated for each lactating ewe and an additional score of 4 was added when signs of arthritis, keratoconjunctivitis or respiratory distress were observed. Based on the resulting scores, clinical indices were determined for each week to estimate the time course of clinical development for each group.

### 2.4. Collection of Milk

Milk yield and quality was recorded each day for both udder halves of each sheep, starting first with the negative control, followed by PLMs and then the PG2 group, first milking the uninfected left udder halves. The daily milk yield was recorded using a measuring cup (measuring cup 1000 mL, WACA^®^, Halver, Germany). At the start of the experiment, the sheep were milked twice a day (morning and evening) using disposable gloves, but during the course of infection, the sheep in which the morning milk yield per udder half declined below 100 mL were not milked in the evening. 

### 2.5. Udder Health, California Mastitis Test (CMT) and Single Cell Counts (SCC)

The California Mastitis Test (CMT) preceded aseptic milk sampling and enabled an estimate of the number of immune cells in the milk. This test was mostly carried out on alternate days with few exceptions, as shown in Table 2. When the test was carried out, the milk from each half of the udder was milked separately in two bowls of the sludge test plate and the milk quality (consistency, color, smell) was assessed. External impurities such as blood or pus were also noted. All the milk was decanted off except about 3 mL, which was mixed with an equal volume of sludge (anionic detergent) in the bowl and the mixture gently swirled. The DNA released from the cells caused a change in consistency, which was assessed as indicated under Table 4. Milk samples from both left and right udder halves were also frozen at −80 °C for determining the more precise quantitative SCC using the Fossomatic method (FOSS ELECTRIC, Hillrod, Denmark). On some inspection days, few sheep did not yield enough milk to perform the CMT or SCC analysis. 

Additionally, an overall scoring system was used to assess udder health (Table 5), in which the California mastitis test (CMT), the consistency of the milk secretion, the severity of pain and the consistency of the udder, and the size of the udder LN were considered. A completely healthy udder scored 4 points, whereas 0 points indicated severe mastitis

### 2.6. Quantitative Analysis of PLMs Recovered from Various Body Sites during Experimental Infection and at Necropsy

The presence of viable mycoplasmas (cfu, colony forming units) was determined in milk, eye, ear and nasal swabs, and in homogenized LNs and organs, as described earlier [10]. The specimens were also diluted 1:10 and 1:100 in Aluotto medium and incubated at 37 °C. 

### 2.7. Euthanasia

At the end of the experiment (15 to 18 days p.i.), sheep were groupwise anesthetized by intravenous (vena jugularis) administration of Xylazine (0.2 mg/kg body weight; Sedaxylan^®^, Eurovet Animal Health BV, Bladel, The Netherlands) and subsequently euthanized by injecting Sodium Pentobarbital (900 mg/10 kg body weight; Release^®^ WDT, Garbsen, Germany) intravenously.

### 2.8. Pathology

The necropsy and the subsequent pathomorphological and -histological examinations were carried out at the Institute of Pathology, University of Veterinary Medicine Vienna, Austria. Features such as body weight and nutritional status were recorded for each sheep before removing the udder tissue for further dissection and analysis. Subsequently, the abdominal and chest cavities were opened to examine the internal organs and LNs for their surface, content, consistency, size, colour, shape, coherence and structure. Samples were removed and stored in 10% neutral buffered formalin, according to Lillie [22], for pathological, and at −80 °C, for bacteriological examination, respectively. Besides internal organs including adrenal glands, the following LNs were examined and sampled: supramammary, subiliac, iliac, medial and lateral retropharyngeal, mediastinal, mandibular, superficial cervical, parotideal, popliteal, jejunal and mesenterial. Additionally, knee and carpal joint tissues and fluids were sampled from both left and right sides for bacteriological examination. For pathohistological sections, the tissue and organ samples were placed in 10% neutral buffered formalin solution for at least 24 h at room temperature for fixation. The samples were then placed in the tissue-Tek infiltration processor (Sanova Pharma Ltd., Vienna, Austria) for embedding in paraffin. Subsequently, 1–2 µm sections were made with the help of a microtome (Leica Biosystems, Nussloch, Germany) and placed on slides in a water bath. Hematoxylin-eosin (HE) staining was carried out for all sections according to the standard protocol described earlier [10] and examined under a light microscope (Olympus BX45, Hamburg, Germany) at 4, 10, 20 and 40× magnification, whereby the nuclei appeared blue and the cytoplasm reddish.

### 2.9. Immunohistochemistry

Histological sections were dewaxed by overlaying with Neo Clear^®^ Solution (Merck, Darmstadt, Germany) and then treated twice with 100% and once with 96% and 70% alcohol each. After rehydration with sterile water, the slides for antigen unmasking were heated with citrate buffer (pH 6.0) in the Lab Vision PT module (Thermo Fisher Scientific, Waltham, MA, USA). In order to block the background staining of the HE-stained preparations, they were incubated for 5 min in a hydrogen peroxide block (Thermo Fisher Scientific, Waltham, MA, USA) and another 10 min in Ultravision protein block (Thermo Fisher Scientific, Waltham, MA, USA). The preparations were then incubated for 30 min with the primary antibody (polyclonal rabbit antiserum against whole cell antigens of *M. agalactiae* type strain PG2; 1:1500) at room temperature, as described earlier [23]. This was followed by incubation with the first antibody booster (Thermo Fisher Scientific, Waltham, MA, USA) for 15 min and, finally, the preparations were treated with an HRP polymer (Thermo Fisher Scientific, Waltham, MA, USA) for 20 min. The preparations were stained for 5 min using a DAB Plus Substrate System (Thermo Fisher Scientific, Waltham, MA, USA). After counterstaining with 1:8 diluted Mayer’s Hematoxylin (Thermo Fisher Scientific, Waltham, MA, USA) for 1 min, the samples were dehydrated in ascending alcohol series (70%, 96% and 100%). Subsequently, the sections were treated with Neo Clear^®^ and Neomount (Merck, Darmstadt, Germany) for microscopic examination.

### 2.10. Statistical Analyses

The statistical analysis of the obtained data was carried out with the program IBM SPSS Statistics Version 22 at the Department of Biomedical Sciences, University of Veterinary Medicine Vienna. In addition to descriptive statistical methods, post hoc tests, Mann–Whitney tests and *t*-tests were used. Differences between individual groups were analyzed by ANOVA and those with a value of *p* < 0.05 were considered significant. 

## 3. Results

### 3.1. Comparison of Clinical Manifestations

Most of the clinical features were assessed using the scoring systems described above. In addition to the analysis and comparison of the individual groups, all PLM groups were also combined and compared with the positive (PG2) and negative (saline) control groups. Although all the calculations did not qualify as statistically significant, especially due to the small number of animals in the individual PLM groups, the results could have important implications.

In contrast to the negative control group, the general condition of the PG2 group and the PLM groups decreased to a variable extent during the test period (Figure S1). As can be seen in Table S1, significant differences were observed between the negative group and the PG2 group between Day 1 and Day 7, Day 11 and Day 13 and Day 15 p.i. On the other hand, the combined PLM group showed significant differences to the negative control group, starting at Day 2 p.i. until the end of the experiment (except for Day 12 p.i.). Strikingly, the PLMW group showed the worst general health over the period from Day 3 to Day 13 p.i., whereas the greatest impact was seen in the PLMY group, whose score dropped from 4 on Day 1 to 1.67 on Day 3 p.i., although stabilizing again during the course of the experiment (Figure S2; Table S2). The PG2 group showed a significant difference compared to the combined PLM group only on Day 14 p.i. (Table S1).

The average body temperature of the infected sheep was mostly in the physiological range (38.5 and 39.5 °C), except for slightly higher temperatures (ranging from 39.6 to 40.2 °C) observed for a majority of sheep between 2 h and 12 h p.i., except for the PLMX group, which demonstrated this increase from Day 1 to Day 5 p.i.

### 3.2. Udder Health, Milk Yield and Quality

Except for the negative control, all sheep developed severe mastitis and transient agalactia in the right udder halves starting from Day 1 p.i. 

**Figure 1:**
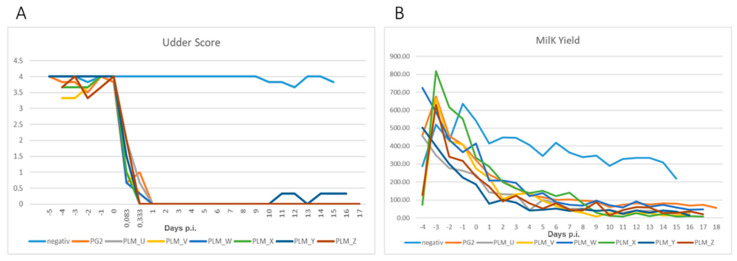
Average values of the udder scores (**A**) and milk yield in mL (**B**) for the eight infected groups of sheep over the test period.

Regarding udder health, all groups, except the negative control group, showed a rapid decline soon after the infection. Both the PG2 and the combined PLM group demonstrated a significant difference in their udder scores (Table 5) compared to the uninfected negative group, starting from 2 h p.i. until the end of the experiment (Figure 1A). The milk yield of the infected right udder halves also decreased very quickly after infection to almost complete drying in all infected animals (Figure 1B), and no significant difference was observed between the milk yield of the PLM and the PG2 groups. (Figure S3). However, the PLM group showed a significant difference compared to the negative group 24 h earlier, i.e., on Day 1 p.i, compared to the PG2 group, for which a significant difference was visible only after Day 2 p.i. (Table S3). The milk yield from the left udders also declined with time, though much less abruptly, but never exhibited complete drying. Additionally, there were less significant differences in the negative group compared to left udder halves. There were no significant differences between the PLM and PG2 groups at any given time (Figure S3).

**Figure 2:**
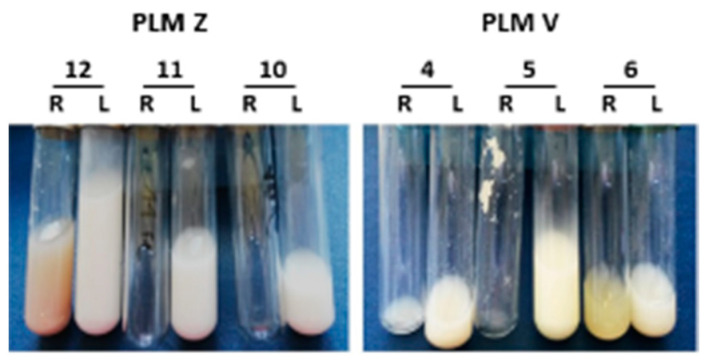
Altered or complete absence of milk from the right udder halves. A representative picture of milk secretions obtained on Day 17 p.i. (PLMZ) and Day 13 p.i. (PLMV), whereby left udder (L) milk samples appear normal but the yield from right udders (R) is either absent (sheep 10 and 11), severely reduced (sheep 4) or is abnormal and discolored, varying from watery yellow (sheep 6), to flaky (sheep 5) to reddish due to the presence of red blood cells (sheep 12).

Not just the quantity, but the quality of the milk from the right udder halves of infected sheep also showed significant deterioration compared to the left udder halves (Figure 2). If at all present, the milk secretions were either watery greyish to yellowish, sometimes containing flakes and clots, or were reddish due to the presence of blood cells. Figure 2 demonstrates some of these abnormal milk phenotypes from the right udder halves of PLMZ- and PLMV-infected sheep on Day 17 and Day 13 p.i., respectively, when the milk from left udder halves appeared mostly normal.

### 3.3. M. agalactiae Excretion in Milk: PLMU Is Impaired in Initial Host Colonization and Multiplication at the Site of Infection

**Figure 3:**
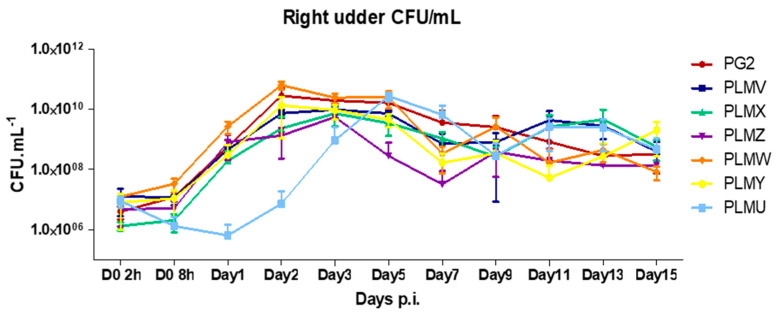
*M. agalactiae* load in milk samples obtained from the inoculated right udder halves of ewes experimentally infected with 10^9^ cfu of six different Vpma PLMs (PLMV, PLMX, PLMZ, PLMW, PLMY and PLMU) and type strain PG2. Mean log_10_ cfu values of mycoplasmas per milliliter of milk from three sheep in each group are plotted and bars represent standard error of the mean.

*Mycoplasmaagalactiae* was re-isolated from the milk of infected sheep right after 2 h of infection until the end of the experiment (Figure 3). For all infection groups, except PLMU, the mycoplasma counts started increasing right after 2 h p.i. and the highest bacterial loads were observed between Day 2 and Day 3 p.i., ranging from 5.52 × 10^9^ cfu/mL to 6.0 × 10^10^ cfu/mL. Unlike other infection groups, PLMU showed a rapid decline in the first 24 h p.i., whereby only 8% of the initial inoculum could survive in milk secretions. Thereafter, PLMU counts started increasing and the highest PLMU loads were detected only at Day 5 p.i (Figure 3); that is 2–3 days later than the other six infection groups, clearly pointing towards an initial defect in colonization and multiplication.

Milk samples from left udders were found positive for mycoplasmas only in a few sheep. This also started only at around one week after initial infection and persisted until the end of the infection trial (Figure S4). Two sheep each from the PLMV and PLMX infection groups, and one from PG2 group, showed a similar trend, with the highest counts witnessed between Day 11 and Day 13 p.i. For PLMZ, one sheep exhibited a single sporadic positive result on Day 1 p.i. and was found to be negative on all subsequent days. The earlier *M. agalactiae* intramammary infection trials had yielded sporadic re-isolations from the milk of left uninoculated udder halves [13] with much lower loads compared to the current study, except for the PG2-infected sheep. As observed during earlier mycoplasma mastitis cases, the excretion of mycoplasmas in milk showed a biphasic trend with alternate high and low phases of recovery, likely due to immune modulation [13,24].

### 3.4. Mycoplasma Re-Isolations from Organs and Lymph Nodes: PLMV Shows Maximum Numbers of Positive Tissues

Analysis of 40 different necropsied organs and LNs was made for each sheep to check for the presence of *M. agalactiae*, and the results are provided in Table 6. The right udder halves and the right supramammary lymph nodes were almost always positive for all infected sheep (Table 6). Unlike previous experimental intramammary infections made by our group [10,13,23,25,26], the current infection trial did not lead to a prominent systemic spread of *M. agalactiae* to organs and lymph nodes distant from the site of inoculation, except for a few cases. In this context, sporadic re-isolations were obtained from the kidneys, brain, LN cervicalis superficialis, popliteal LNs, medial retropharyngeal LNs, and, interestingly, also from the adrenal glands and sub-iliac LN. The iliac LN was a common site for re-isolations. In all the above cases, as expected, most of the isolations were from the right side of the body and only sporadically from the left side, except that the left udder halves, which were positive for mycoplasmas in almost all ewes. For unknown reasons, PG2-infected sheep showed variable re-isolation counts in the two different parts of the infection trial. 

An important result was that the maximum number of re-isolations, (a total of 23 positive re-isolations) were obtained from the PLMV infection group, closely followed by PLMW. These results could, again, be very well correlated to the highest adhesion and invasion potential of PLMV and PLMW in in vitro adhesion assays [19].

### 3.5. Immune Cells in the Milk

**Figure 4:**
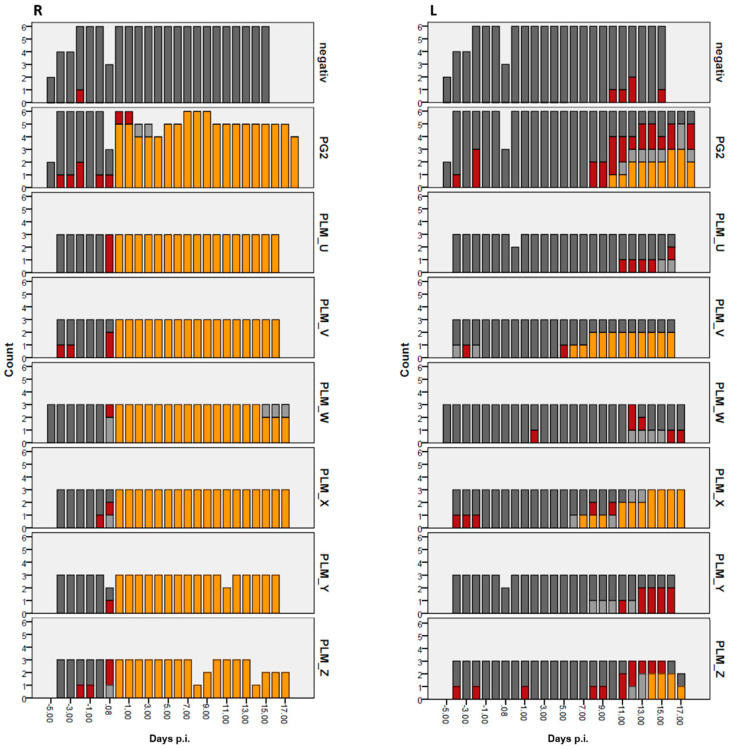
Graphic representation of the California Mastitis Test (CMT) scores of the infected sheep groups and the negative control group. The right panel denotes milk from the left udder halves (**L**) and the left one denotes milk from right udder halves (**R**). Assessment was primarily made according to Table 4 to demonstrate the degree of gel formation: dark grey [−/(+)], red [+], light grey [++], yellow [+++].

Bacterial infections of the udder often lead to varying degrees of inflammation. The presence and relative quantity of immune cells in milk secretions is often a good indicator of the severity of mastitis. *M. agalactiae* is known to initiate a dynamic innate immune response at the site of infection, often leading to an adaptive response [27,28]. At a daily level, this was estimated by CMT (Table 4), whereby the right udder milk samples exhibited high-grade viscosity and clot formation in all infected sheep, even before the completion of 24 h p.i., until the end of the experimental trial (Figure 4R). CMT analysis of left udder milk samples demonstrated positive gel-formation results only after Day 7–Day 9 p.i., as seen for PLMX-, PLMV-, PLMZ- and PG2-infected sheep (Figure 4L).

**Figure 5:**
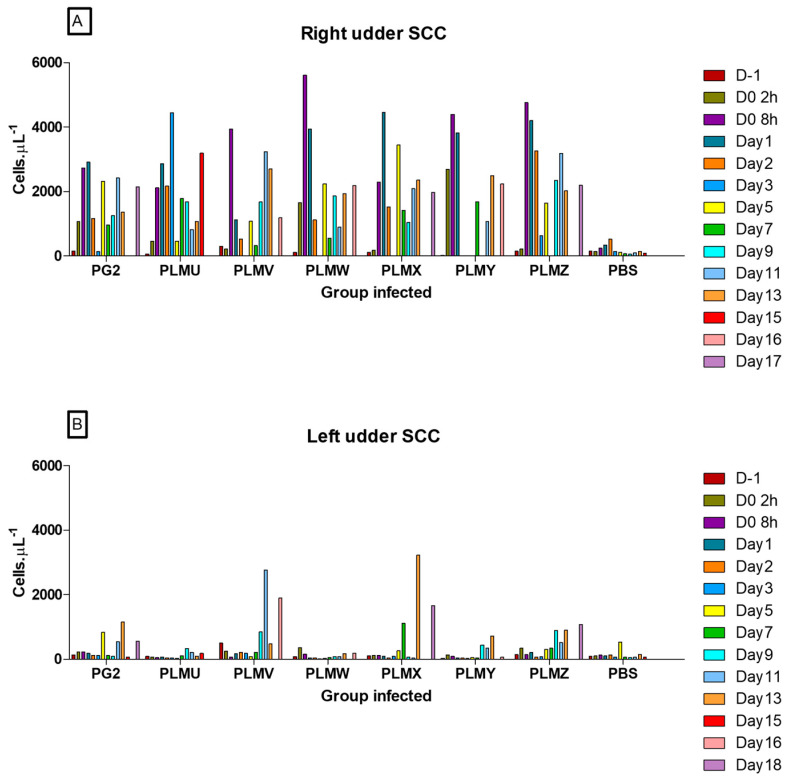
Single Cell Counts in milk from right (**A**) and left (**B**) udder halves of sheep infected with different PLMs and the positive (PG2) and negative (PBS) controls.

In addition to the daily quality estimation by CMT, quantitative SCC counts in milk were measured at regular intervals. Like other mycoplasma lipoproteins, Vpmas could serve as important immunomodulins, inducing the recruitment of granulocytes, lymphocytes, macrophages, and cytokines, which, in turn, could affect the quantity and quality of milk. We expected that any differences in the pathogenic and immunomodulatory potentials of the six Vpmas would also be reflected in the SCC of milk. However, a total lack or poor quality of milk for some sheep on some days, especially towards the end of the experiment, hampered precise evaluations and comparisons by this method in a statistically significant manner. Nevertheless, it could be clearly observed that the cells in the right udder halves showed a rapid increase after experimental infection, whereas the number of cells in the left udder milk samples increased with a delay, but never to the same extremes (Figure 5). Amongst the right udder milk samples, one of the PLMZ-infected sheep (No. 11) exhibited the highest cell count of 6378 cells/µL at 8 h p.i., whereas a PG2-infected sheep (No. 13) showed a cell count of 4112 cells/µL on Day 13 p.i. as the highest count in the left udder milk samples. These SCC values are similar to those obtained during previous *M. agalactiae* experimental infections of sheep [13].

### 3.6. Pathological Anatomical Differences: PLMV and PLMX Show Significant Hyperplasia of the Left Udder Lymph Nodes

The nutritional status of all experimental animals was assessed to be good, except for the PG2-infected sheep No. 13, which was slightly below average. Amongst the pathological-anatomical sections analyzed, changes in the udder lymph nodes were evaluated using statistical data analysis methods. 

Hyperplastic changes in the right and left udder lymph nodes were assessed as minor (1.00), mild-moderate (1.50), moderate (2.00), moderate-high (2.50) and severe (3.00). The results were summarized for all animals in the groups and total frequencies calculated. The results pertaining to the hyperplasia of the right and left udder lymph nodes are enlisted under Table S4A and S4B, respectively.

In comparison to the other infection groups, PLMV and PLMX were the only groups that showed significant differences in the hyperplasia of the left udder lymph nodes (Table S4). This was because these groups had the most positive diagnosis and showed highly or moderately enlarged left udder lymph nodes.

### 3.7. Histopathology: PLMV and PLMX Show Inflammatory Cells and Purulent Galactophoritis on the Left Side

A high-grade alveolar pulmonary edema was found in all sheep infected in the first round of experimental infections, and two sheep from the second part, namely one PLMV-infected sheep (No. 4) and one sheep from the negative control group (No. 2). On the other hand, a moderate or low-grade alveolar pulmonary edema was diagnosed in one or two sheep of all infection groups of the second infection trial. The descriptive statistics showed that the animals in the first part of the experiment were more severely affected, although no correlations could be drawn with mycoplasma infection, as these features were also observed in the negative control group sheep of both infection trials. 

Hyperemia of the lungs was observed in nine lung sections (distributed across all infection groups except the PG2 group) from the first trial, and one sheep from the second trial. Other organs such as the liver and spleen also showed hyperemia. A few animals, distributed across all groups showed evidence of low-grade, and rarely of moderate, hyperemia. Six animals showed non-purulent inflammatory cells on the myocardium, with one section each from the negative control group, one from the PLMW, PLMV and the PLMX groups, and two sections from the PG2 group demonstrating focal myocarditis.

All groups, with the exception of the negative control group, showed a non-purulent interstitial mastitis and purulent galactophoritis in the right udder halves. With the exception of PLMZ-infected sheep No.12, all other animals showed a moderate to highly severe non-purulent interstitial mastitis. The purulent inflammation of the milk-bearing ducts was slight or moderate on the right udder halves. The negative control group, as well as one sheep from each of the groups PG2, PLMX and PLMZ, showed no inflammation. In contrast to the right udder halves, inflammatory cells could hardly be found in the interstitial tissue on the left side. Only two sheep each from the PLMV and PLMX groups showed low-level non-purulent inflammation. These four histological sections were also the only ones to have a slightly purulent galactophoritis on the left side.

Additionally, proliferation of the interstitial connective tissue associated with mastitis was more pronounced in the infected right udder halves compared to the uninfected left udder halves. Two sheep from the negative control group (of the first part of the trial) also showed a similar change in the right udder halves. The left side demonstrated such results sporadically in each group, which, with the exception of one outlier, never exceeded a moderate level.

In addition, micro abscesses were observed in the right glandular parenchyma in one sheep each from the PG2, PLMU, PLMV, PLMX and PLMZ infection groups. 

The exposed follicular centers in many of the examined lymph nodes were striking. Here, a less eosinophilic colored germinal center was observed in the secondary follicles. This was especially significant in the PLMV group where 14 lymph nodes demonstrated such a change, including all right udder lymph nodes. The remaining groups were affected differently, and it could be seen that the lymph nodes of the test groups were less affected in the first part of the infection trial, with only four clearings compared to 39 total clearings in the second part, whereby most of these belonged to the right udder lymph nodes (Table 7).

### 3.8. Immunohistochemistry: PLMV Shows Significantly Higher Spread to Tissues and Organs Away from Inoculation Site

Immunohistochemistry was performed on the necropsied samples that were found positive for *M. agalactiae* isolations during bacteriological examinations. The two analysis methods were compared and statistically analyzed. The test results were checked on the basis of the individual groups and also by combining the six PLM groups into one group and compared with the positive control. Comparison between the results of immunohistochemistry and bacteriology yielded significant differences, whereby only 71 samples were found positive via immunochemistry in contrast to 129 positives by culturing. These results of bacteriological examination and immunohistochemistry when plotted graphically lead to the ideal placement of points on a straight line (Figure S5).

Table 8 enlists the tissues in which *M. agalactiae* was most frequently detected via immunohistochemistry, the right udder halves and right supramammary LNs being the toppers in the list. Occasional positive results were observed for medial retropharyngeal LNs (right/left), mandibular LNs (right), LN cervicalis superficialis (right/left), mediastinal LNs and popliteal LNs (right/left).

Interestingly, the PLMW group did not show any positive results in the infected udder halves, although the suprammammary LN on the right side were positive for all ewes. Even in the PLMU group, *M. agalactiae,* could be detected in the right udder halves only in one sheep. This was in contrast to the bacteriology results, which detected *M. agalactaie* in the right udder halves and/or right supramammary LNs in all infected animals (Table 6).

**Figure 6:**
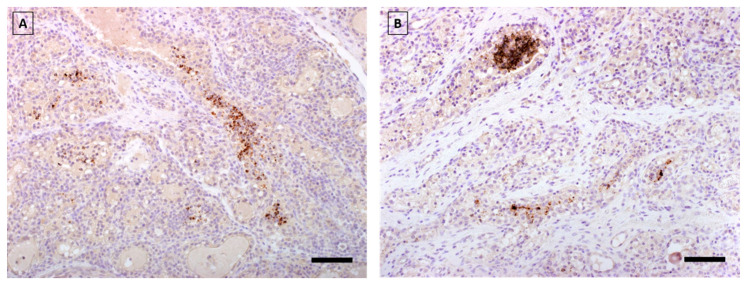
Immunohistochemical detection of *M. agalactiae* in left udder samples of PLMV-infected (**A**) and PLMX-infected (**B**) sheep. *M. agalactiae* specific brown reaction products are observed using anti-*M. agalactiae*-specific rabbit polyclonal antibodies (bar = 80 µm).

The spread of *M. agalactiae* from the infected right udder halves to the uninfected left udder halves was confirmed by immunohistochemistry in two sheep from PLMV, and one sheep from the PLMX infection group (Figure 6). The same animals also showed the presence of *M. agalactiae* in the left supramammay LNs.

The presence of the pathogen in the neck region was especially prominent in the PLMV group, as *M. agalactiae* could be detected in the medial retropharyngeal LNs (right/left), mandibular LNs (right) and in the LN cervicalis superficialis (right). One of these sheep (no. 6) showed the most positive results. The statistical evaluation of the group comparisons showed that the PLMV group, with the exception of the negative control group, was the only group to show significant differences from the other groups. 

Some of the additional necropsied samples that had shown minimal bacterial growth tested positive for immunohistochemistry signals. These samples included the right lateral pharyngealis LN, kidneys (right and left), adrenal glands (right and left), knee and carpal joint (both left) and synovia of the right carpal joint.

## 4. Discussion

The aim of this study was to determine if intramammary infections of lactating ewes with the six *M. agalactiae* PLMs and the wild type PG2 strain differ in terms of clinical parameters (general condition, internal body temperature, udder health, milk yield, number of cells in milk), pathological changes (pathomorphology, pathohistology, immunohistochemistry) and mycoplasma excretion in milk and re-isolations from necropsied organs and tissues. 

As suggested by Bergonier et al. [29], a febrile phase appeared in all infected groups within 4 h of infection, except for the PLMX group, in which the increase in the internal body temperature was first observed only at Day 2 p.i. A significant decrease in the general well-being of the infected sheep was noticed on Day 1 p.i. in the PG2 group and Day 2 p.i. in the PLM group. The rapidity of the clinical signs was remarkable, especially when compared to 7 to 56 days incubation period for naturally occurring infections [30]. It can therefore be assumed that the rapidly generated clinical changes are due to the experimental setup with the direct intramammary application of a high infection dose.

Clinically significant differences in udder health could only be observed compared to the negative control group. After infection, the udder health deteriorated rapidly, as early as 2 h after inoculation, accompanied by qualitative and quantitative changes in milk secretions, initially on the right side (significant differences of the PG2 group compared to the negative control from Day 2 p.i., and PLM group and the negative group from Day 1 p.i.), and later on the left non-inoculated udder halves (significant differences between the PG2 group and the negative control group on Days 6, 8 and 10 p.i., and between PLM and the negative control group on Days 1, 6, 8, 9, 10, 11 and 13 p.i.), as described earlier [13,29]. 

One of the most important results of this study was the rapid decline in PLMU loads in milk secretions in the first 24 h p.i. in contrast to all other infection groups, which showed an increase in mycoplasma CFU in milk right after 2 h p.i. These results are in direct agreement with an earlier PLMU/PLMY co-challenge study where PLMY completely outcompeted PLMU at the local site of infection [10], especially in the first week of infection. In the latter study, although an equal mixture of PLMU and PLMY was used as inoculum via the intramammary route, PLMU was not detected in the milk of 4 out of 5 inoculated sheep starting from Day 1 p.i. until the end, i.e., Day 26 p.i., and even so, the one sheep that was found positive showed the presence of PLMU only after Day 12 p.i. [10]. This was the first study to indicate the lower in vivo fitness of VpmaU expressors compared to VpmaY expressors in a co-challenge experimental infection. In the current study, we wanted to evaluate the infection profile of VpmaU expressers by using PLMU as sole inoculum in three different lactating ewes to negate any competitive effects of other Vpma phenotypes in the starting inoculum. Indeed, the rapid decline in mycoplasma counts in the milk of PLMU-infected sheep was clearly visible, and a mere 8% of the initial cfu survived by the end of 24 h p.i., indicating an initial colonization and multiplication defect. This was in contrast to all other infected sheep for whom the mycoplasma cfu counts showed a gradual increase right after 8 h p.i (Figure 3). The lower colonization potential of PLMU correlated very well with the in vitro adhesion assays, which demonstrated VpmaU as the least adherent of all Vpma adhesins [19]. However, it is also possible that the PLMU expressing VpmaU lipoprotein is more susceptible to phagocytic killing [31], as seen in the smaller variants of the homologous Vsa lipoproteins of *M. pulmonis* [6].

Another interesting finding was the maximum number of mycoplasma re-isolations obtained in the PLMV infection group. This result is in accordance with the in vitro cell culture studies, whereby PLMV showed the highest adhesion and invasion potential compared to the other five PLMs [19]. Additionally, VpmaV bears adhesive epitopes, which had been earlier shown to be important for adhesion of Vsp proteins of *M. bovis* [14]. 

In contrast to the reports of Bergonier et al. [29], describing catarrhal to parenchymatous mastitis caused by *M. agalactiae*, the current intramammary infection led to a non-purulent interstitial mastitis and purulent galactophoritis, as also observed during our previous studies [32]. The presence of inflammatory cells in the interstitium and milk ducts of the left udder halves of selected sheep, predominantly in the PLMV and PLMX groups, could be indicative of differences in the systemic spread and virulence of the corresponding Vpmas.

The observed hyperplasia of the right supramammary LN has been described earlier by Bergonier et al. [29]. Importantly, the comparison of the enlarged udder lymph nodes of various groups demonstrated a significant difference in the case of PLMV and PLMX for the left supramammary LN, again pointing towards the increased virulence of these two Vpma phase-locked mutants of *M. agalactiae*.

The “lightening”, as observed histologically in the secondary follicles of the LN, means lymphocyte depletion and suggests an increased reaction of the LN to *M. agalactiae*. These reactions were the strongest for the right supramammary LNs, a result that could easily be attributed to the intramammary inoculation of *M. agalactiae* into the right udder halves. A significantly higher number of such histological changes were reported for the PLMV, PLMX and PLMZ infection groups (a total of 39 detections), indicating differential immune characteristics and virulence potential of Vpma V-, X- and Z-expressors found in the corresponding PLMs.

Interestingly, *M. agalactiae* could not be detected immunohistochemically in the right udder halves of the PLMU and PLMW groups. This could indicate clearance of the pathogen at the primary site of infection and migration to the regional lymph nodes. Detection of *M. agalactiae* in the pharynx of PLMV-infected sheep could be due to an oral infection or the oropharyngeal settlement of the pathogen after systemic spread. A possible oral infection could be explained by mutual suckling or by environmental contamination with subsequent oral ingestion of the pathogen. An infection of the contralateral, uninfected left udder halves could only be detected immunohistochemically in the PLMV and PLMX groups (Figure 6). This could, in turn, be caused by contamination of the left teat during the milking process, or by environmental contamination with subsequent ascending infection. Haematogenic spread of the pathogen and infection of the left half of the udder via the blood-udder barrier would also be possible.

Altogether, the immunohistochemical examination and the detection of the pathogen via bacteriological culture methods demonstrated significant differences. Culture is regarded as the gold standard of mycoplasma diagnosis [33] as it can lead to the isolation and enrichment of mycoplasmas even if they are present in minimal amounts in the test material. As expected, the immunohistochemistry method did not reach the sensitivity of the cultural pathogen detection. Despite different results, both methods demonstrated the systemic spread of the pathogen to distant organs, lymph nodes, joints and glandular tissue. *M. agalactiae* was most frequently detected in tissue structures outside the primary site of infection in PLMV and PLMX infection groups. However, no indication of an increased systemic spread of the pathogen was evident in these two groups based on clinical examinations.

## 5. Conclusions

Although no significant differences between the wildtype strain PG2 and the PLMs could be detected clinically, the study proves that all six individual Vpma expressers of *M. agalactiae* type strain PG2 can initiate an infection, leading to rapid mastitis within 24 h p.i., and that for all PLM groups, high loads of mycoplasmas were excreted in milk until the end of the experiment, comparable to PG2 strain. Most interestingly, unlike all other PLMs and PG2 strain, PLMU demonstrated an initial defect in host colonization and multiplication, a result that is in agreement with previous in vitro and in vivo studies, where PLMU showed the lowest cytadhesion capability and was almost completely outcompeted during a PLMU and PLMY coinfection study. Additionally, the maximum number of mycoplasma re-isolations obtained for the PLMV group is in accordance with the highest adhesion and invasion potential of PLMV observed during in vitro assays. Furthermore, pathological examinations showed significant differences regarding hyperplasia of the udder lymph nodes in the groups PLMV and PLMX, compared to the other infected groups. This, together with their spread to other LNs and organs away from the infection site, including the left supramammary LNs and/or left udders, indicates the higher virulence of the VmpaV and VpmaX expressers. 

The inability to vary the Vpma expression phase does not hamper the initiation of infection, or colonization and multiplication of the pathogen at the site of inoculation, for all six PLMs. This is the first study where all isogenic expression variants of a large mycoplasma multigene family have been individually tested in in vivo infection studies in their natural host to rule out non or impaired infectivity, and to elucidate the role and significance of individual members in infection.

## Figures and Tables

**Table 1:** Part 1 (A) and Part 2 (B) of experimental *M. agalactiae* infections of ewes carried out via the intramammary route.

Animal Groups	Positive Control Group	Negative Control Group	Mutant Group
(A)			
**Inoculum**	*M. agalactiae* type strain PG2	sterile & pyrogen free saline	*M. agalactiae* Vpma
			phase-locked mutants
			PLMU, PLMY and PLMW
**Inoculum Size**	10^9^ CFU	-	10^9^ CFU
**Infection Route**	intramammary	intramammary	intramammary
	via right teat	via right teat	via right teat
**Animals**	3 lactating ewes	3 lactating ewes	3 lactating ewes/PLMU
			3 lactating ewes/PLMY
			3 lactating ewes/PLMW
			=9 animals
(B)			
**Inoculum**	*M. agalactiae* type strain PG2	sterile & pyrogen free saline	*M. agalactiae* Vpma
			phase-locked mutants
			PLMX, PLMZ and PLMV
**Inoculum Size**	10^9^ CFU	-	10^9^ CFU
**Infection Route**	intramammary	intramammary	intramammary
	via right teat	via right teat	via right teat
**Animals**	3 lactating ewes	3 lactating ewes	3 lactating ewes/PLMX
			3 lactating ewes/PLMZ
			3 lactating ewes/PLMV
			=9 animals

Necropsy 15–18 Days post infectionem (p.i.).

**Table 2:** Monitoring the clinical course of infection and sample collection.

Day p.i. ^1^	Clinical Examination ^2^	Feed Intake, Milk Yield	Body Temp ^3^	Blood ^4^	Milk ^5^	Swabs ^6^
D-5	X	X	X		X	
D-4	X	X	X			
D-3	X	X	X			
D-2	X	X	X			
D-1	X	X	X	X	X	X
D0	X	X	X			
D0 2 h p.i.	X		X		X	
D0 4 h p.i.			X	X		
D0 8 h p.i.	X	X	X		X	
D0 12 h p.i.			X	X		
D1	X	X	X	X	X	
D2	X	X	X	X	X	
D3	X	X	X		X	
D4	X	X	X			
D5	X	X	X		X	
D6	X	X	X			
D7	X	X	X		X	X
D8	X	X	X			
D9	X	X	X	X	X	
D10	X	X	X			
D11	X	X	X		X	X
D12	X	X	X			
D13	X	X	X		X	
D14	X	X	X			
D15	X	X	X	X	X	X
D15–18	Euthanasia and pathological examination					

^1^ p.i.: post infectionem. ^2^ according to the specifications of clinical propaedeutics. In addition, a weekly clinical index was created to quantify clinical symptoms during the experiment (Table 3); milk production and consistency (Table 4), as well as the clinical status of the udder (4-normal udder, 0-severe mastitis) was evaluated (Table 5). The points were added together for each sheep. ^3^ inner body temperature (°C), rectal measurement. ^4^ collection of blood from the *Vena jugularis* using Vacutainer. ^5^ aseptic sampling of milk for bacteriological and single cell count analyses. ^6^ swab sampling (eye, ear, nose and vagina).

**Table 3:** Assessment of general well-being after *M. agalactiae* infection.

Appetite	General Behavior	Conjunctiva	Pulse	Breathing	Auscultation Lungs	Points	General Condition
+++	Calm and attentive	Pale pink–moderately red/anemic	56–100	16–30	Slightly a.v.	4	not affected
++	Slightly changed	moderately reddened	101–124	21–45	moderately a.v.	3	slightly impaired
+	Moderately changed	highly reddened	>125	46–60	moderate to highly a.v.	2	moderately impaired
0	Highly changed	highly reddened	>125	>60	highly a.v.	1	highly impaired

a.v. = aggravated vesicular.

**Table 4:** Assessment key for indirect cell count determination using the California mastitis test [21].

Assessment	Test Pattern	Cells/mL of Milk
−/(+)	Liquid/Slight streaking	<150,000 cells/100,000–250,000
+	High streaking	200,000–700,000
++	Gel formation	500,000–1,500,000
+++	Gel and clog formation	>1,000,000

Physiological cell count in lactating sheep milk: 10,000 to 100,000 cells/mL milk.

**Table 5:** Assessment key for udder health.

CMT	Pain	Milk Secretion	Udder Consistency	Lymph Node Enlargement	Points	Mastitis
-	-	-	se; sebu	-	4	no
+/+++	-	-	se; sebu	-	3	sub-clinical
+/+++	-	slight to moderately changed	se; sebu	-	2	mild
+/+++	−/+	Slight to moderately changed	se; buco; seco	enlarged	1	moderate
++/+++	++/+++	Slight to highly changed	co	enlarged	0	severe

CMT = California Mastits Test; se = soft elastic; sebu = soft elastic to bulging; buco = bulging to. coarse elastic; seco = soft elastic to coarse; co = coarse.

**Table 6:** Bacteriological examination of lymph nodes and organs from sheep inoculated by intramammary route with 10^9^ viable cfu of *M. agalactiae* wild type strain (PG2) or Vpma phase locked mutants (PLMs) and necropsied at Day 15–18 p.i.

**Part I**												
**Infection Group**	**PG2**			**PLMW**			**PLMY**			**PLMU**		
**Organ/Tissue**	**Sheep** **4**	**Sheep** **5**	**Sheep** 6	**Sheep** **7**	**Sheep** **8**	**Sheep 9**	**Sheep** 10	**Sheep 11**	**Sheep 12**	**Sheep** **13**	**Sheep** **14**	**Sheep** **15**
Supramammary LN Left	+		+	+	+		+	+		+	+	+
Supramammary LN Right	+	+	+	+	+	+	+	+	+	+	+	+
Mandibular LN Left				+								
Iliac LN Left						+						
Iliac LN Right				+		+	+			+		+
Parotidial LN Left							+					
Popliteal LN Right		+										
Med retropharyngeal LN				+								
Udder Left	+	+	+	+	+	+	+	+	+	+	+	+
Udder Right	+	+	+	+	+	+	+	+	+	+	+	+
Kidney Right	+				+	+						
Brain	+											
Carpel Joint tissue Left									+			
Knee joint Left	+				+							
Subiliac LN Left												+
Subiliac LN Right	+	+			+				+	+	+	+
Adrenal gland Left					+							
**Total Isolations**	8	5	4	7	8	6	6	4	5	6	5	7
	**PG2 = 17**			**PLMW = 21**			**PLMY = 15**			**PLMU = 18**		
**Part II**												
**Infection Group**	**PLMV**			**PLMX**			**PLMZ**			**PG2**		
**Organ/Tissue**	**Sheep** **4**	**Sheep** **5**	**Sheep** **6**	**Sheep** **7**	**Sheep** **8**	**Sheep** **9**	**Sheep** **10**	**Sheep** **11**	**Sheep 12**	**Sheep** **13**	**Sheep** **14**	**Sheep** **15**
Supramammary LN Left	+		+	+	+	+			+			
Supramammary LN Right	+	+	+	+	+	+	+	+	+	+	+	+
Iliac LN Left			+			+						
Iliac LN Right	+		+	+		+						
LN Cerv superficialis Left									+			
LN Cerv superficialis Right			+									
Mediastinal LN							+					
Med retropharyngeal LN L		+	+									
Med retropharyngeal LN R	+	+										
Lateral retropharyngeal LN		+										
Udder Left	+	+	+	+	+							
Udder Right	+	+	+		+	+	+	+	+	+	+	+
Kidney Left						+	+					
Kidney Right							+					
Carpel joint fluid right			+									
Subiliac LN Right	+			+					+			
Adrenal gland Left	+									+		
Adrenal gland Right						+						
**Total isolations**	8	6	9	5	4	7	5	2	5	3	2	2
	**PLMV = 23**			**PLMX = 16**			**PLMZ = 12**			**PG2 = 7**		

**Part I:** +: positive for *M. agalactiae* isolation. The following LN/tissue samples were negative for *M. agalactaie* isolation in all above tested sheep and hence not placed in this part: Mandibular LN R, Mesenterial LN, Mediastinal LN, Cervicalis superficialis LN L/R, Parotidial LN R, Jejunal LN, Lateral retropharyngeal LN, Popliteal LN L, Carpel Joint Tissue R; Carpel Joint Fluid L/R, Knee joint R, Knee Joint Fluid L/R, Adrenal gland R, Heart, liver, spleen, Kidney L, Lung L/R. **Part II:** +: positive for *M. agalactiae* isolation. The following LN/tissue samples were negative for *M agalactaie* isolation in all above tested sheep and hence not placed in this part: Mesenterial LN, Parotidial LN L/R Mandibular LN L/R, Popliteal LN L/R, Lung L/R, Subiliac LN L, Liver, Heart, Brain, Spleen, Uterus/fallopian tubes; Jejunal LN; Carpel Joint tissue L/R; Carpel Joint Fluid L, Knee Joint L/R; Knee Joint Fluid L/R.

**Table 7:** Sum of thinning of secondary follicles in the respective *M. agalactiae* infection and control groups.

Group	Total Clearings of Secondary Follicles
Negative Control	3
PG2 (positive control)	5
PLM W	1
PLM Y	2
PLM U	3
PLM V	14
PLM X	6
PLM Z	11

**Table 8:** Tissues demonstrating most frequent *M. agalactiae* detections via immunohistochemistry.

Tissue	Udder Right	Udder Left	Supramammary LN Right	Supramammary LN Left	Subiliacus LN Right	Iliacus LN Right
**Total positive** **detections**	18	3	19	6	10	5

## Data Availability

All relevant data are already present in this manuscript or in the Supplementary Materials.

## Supplementary Materials

The following supporting information can be downloaded at: https://www.mdpi.com/article/10.3390/microorganisms10040815/s1, Figure S1: Average values of general well-being of the sheep based on Table 3. PG2 (light grey)- and PLM (black)-infected sheep groups are shown in comparison to the uninfected negative control (red) group. * Individual PLM groups combined to form a single PLM group for analysis; Figure S2: Average values of the general well-being of the eight infected groups of sheep over the test period based on Table 3; Figure S3: Average milk yield (in mL) of the right udder halves (A) and left udder halves (B) of the PG2 (light grey), and PLM (black)-infected sheep groups as compared to the uninfected negative control (red) group; Figure S4:. *M. agalactiae* load in milk samples obtained from the left udder halves of ewes experimentally infected with 10^9^ cfu of six different Vpma PLMs (PLMV, PLMX, PLMZ, PLMW, PLMY and PLMU) and type strain PG2; Figure S5: Correlation between the results of *M. agalactiae* bacteriological examination by culture method and by immunohistochemistry (IHC) using polyclonal rabbit antiserum against whole cell antigens of *M. agalactiae* type strain PG2.; Table S1: Calculation of significant differences in the general well-being (significant differences in bold) of the experimental sheep; Table S2: Course of the average internal body temperature (°C) over the test period; Table S3: Calculation of significant differences in the milk yield from the infected right udder halves between the groups (significant differences in bold); Table S4: Hyperplasia of the right (A) and left (B) udder lymph node.
