# Supplementary material for: Sheep Infection Trials with ‘Phase-Locked’ Vpma Expression Variants of Mycoplasma agalactiae—Towards Elucidating the Role of a Multigene Family Encoding Variable Surface Lipoproteins in Infection and Disease
Source: Microorganisms. 2022 Apr 14;10(4):815. doi: 10.3390/microorganisms10040815 (PMC9025108; doi:10.3390/microorganisms10040815)
Supplement: Supplementary file 1 [file microorganisms-10-00815-s001.zip › microorganisms-1640850-Supplymentary Figures.pdf]

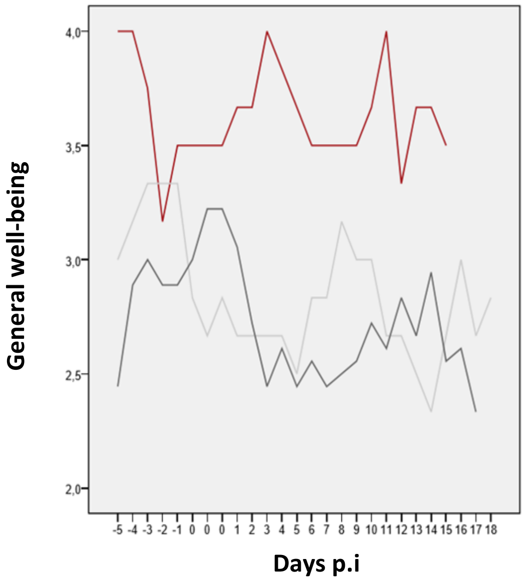


**Figure S1**. Average values of general well-being of the sheep based on Table 3. PG2 (light grey)- and PLM (black)-infected sheep groups are shown in comparison to the uninfected negative control (red) group. *Individual PLM groups combined to form a single PLM group for analysis.

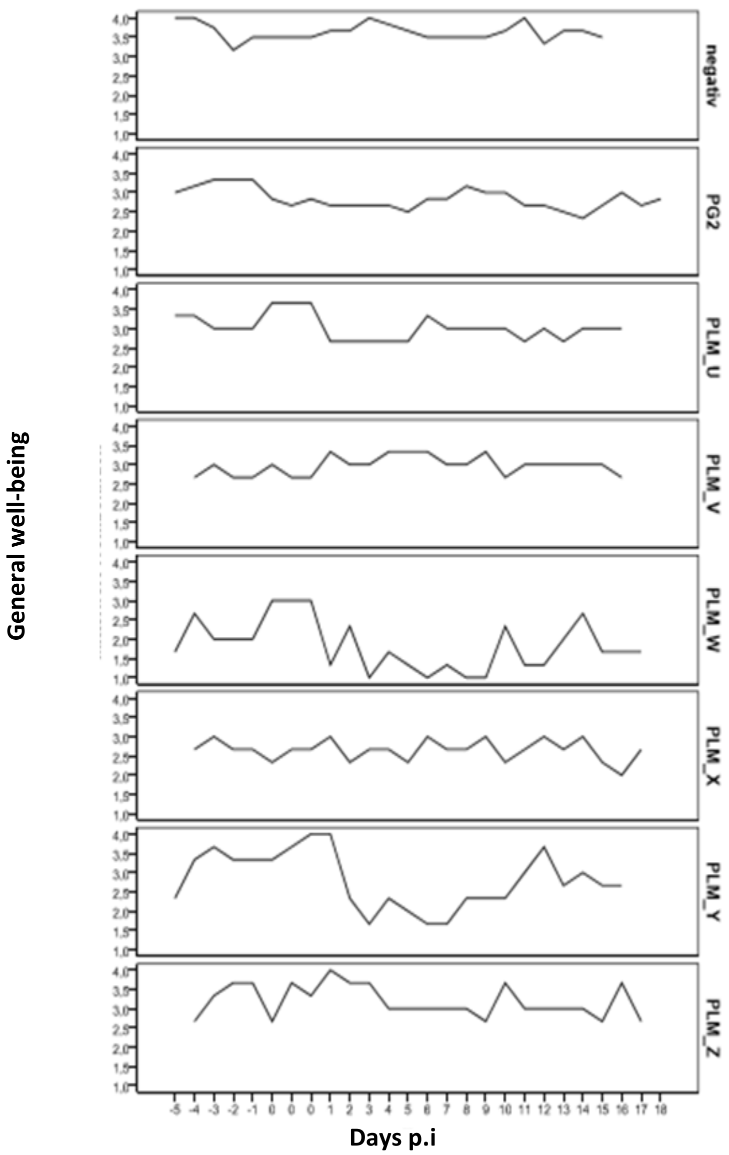


**Figure S2.** Average values of the general well-being of the eight infected groups of sheep over the test period based on Table 3.

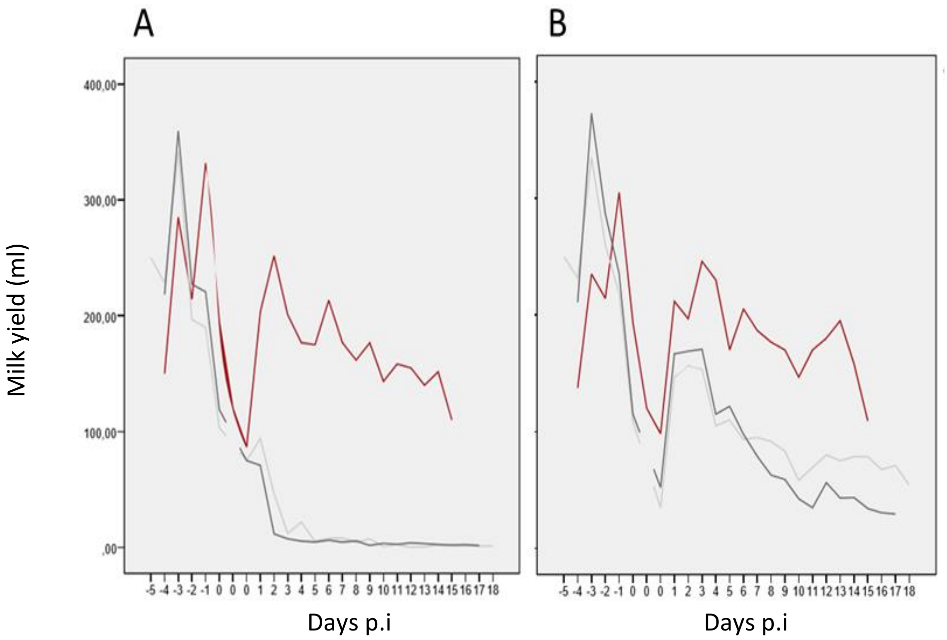


**Figure S3.** Average milk yield (in ml) of the right udder halves (A) and left udder halves (B) of the PG2 (light grey), and PLM (black)-infected sheep groups as compared to the uninfected negative control (red) group.*Individual PLM groups combined to form a single PLM group for analysis

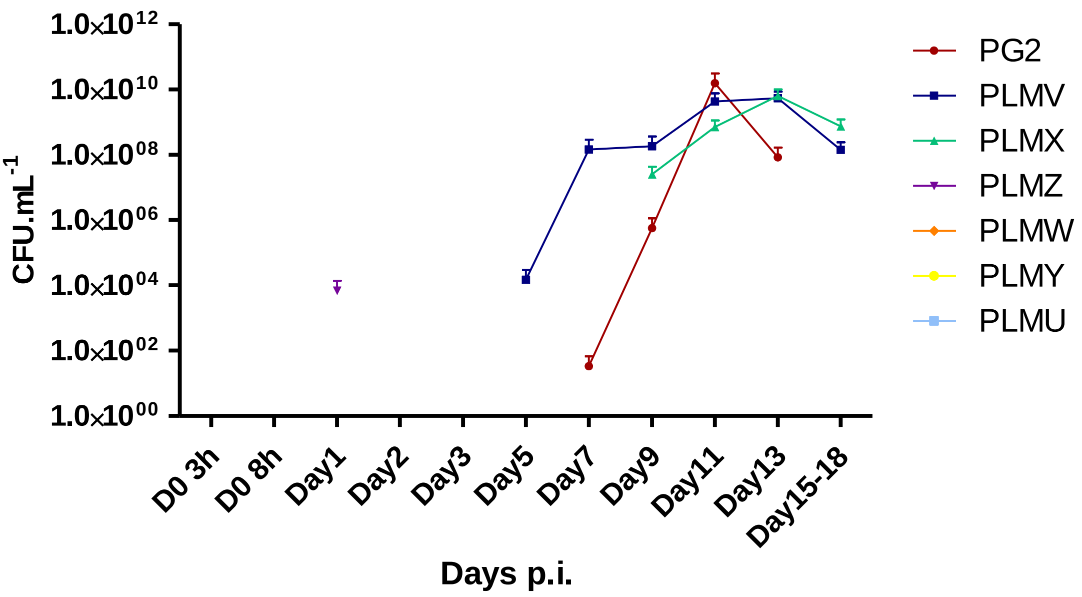


**Figure S4.** *M. agalactiae* load in milk samples obtained from the left udder halves of ewes experimentally infected with $10^9$ cfu of six different Vpma PLMs (PLMV, PLMX, PLMZ, PLMW, PLMY and PLMU) and type strain PG2. Mean $\log_{10}$ cfu values of mycoplasmas per milliliter of milk from three sheep in each group are plotted, and bars represent standard error of the mean.

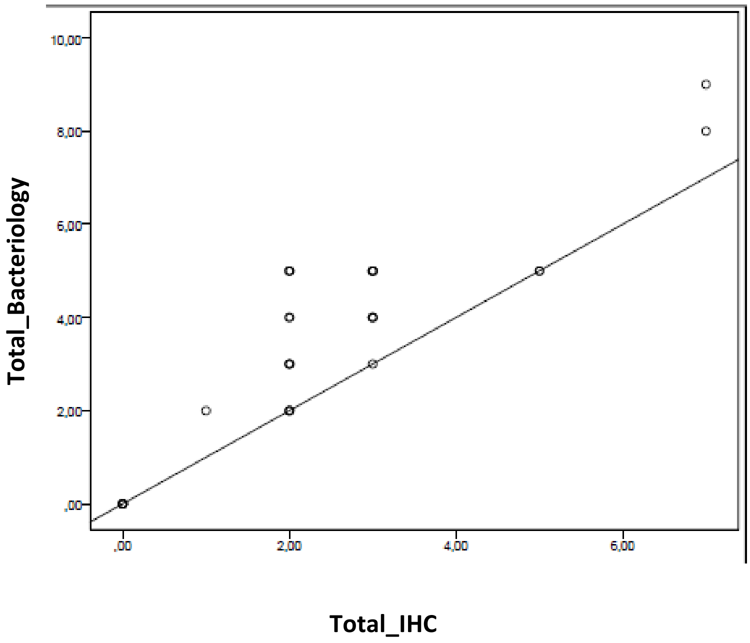


**Figure S5.** Correlation between the results of *M. agalactiae* bacteriological examination by culture method and by immunohistochemistry (IHC) using polyclonal rabbit antiserum against whole cell antigens of *M. agalactiae* type strain PG2.
