# Supplementary material for: Sheep Infection Trials with ‘Phase-Locked’ Vpma Expression Variants of Mycoplasma agalactiae—Towards Elucidating the Role of a Multigene Family Encoding Variable Surface Lipoproteins in Infection and Disease
Source: Microorganisms. 2022 Apr 14;10(4):815. doi: 10.3390/microorganisms10040815 (PMC9025108; doi:10.3390/microorganisms10040815)
Supplement: Supplementary file 1 [file microorganisms-10-00815-s001.zip › microorganisms-1640850-Supplymentary Tables.pdf]

Table S1. Calculation of significant differences in the general well-being (significant differences in bold) of the experimental sheep. Significance was calculated using the Mann-Whitney test. An error probability of p <0.05 was assumed to be significant.

Day	Negative Control/ PG2T	Negative Control/ PLM	PG2T/ PLM
-5	0,121	**0,018**	0,465
-4	**0,014**	**0,006**	0,334
-3	0,221	0,052	0,353
-2	0,863	0,470	0,189
-1	0,476	0,082	0,189
0	**0,043**	**0,092**	0,380
0, 2 h p.i.	0,116	0,368	0,210
0, 8 h p.i.	0,080	0,306	0,163
1	**0,014**	0,170	0,200
2	**0,014**	**0,009**	0,821
3	**0,007**	**0,001**	0,725
4	**0,006**	**0,003**	0,826
5	**0,011**	**0,004**	0,912
6	**0,043**	**0,028**	0,664
7	**0,043**	**0,005**	0,280
8	0,241	**0,013**	0,094
9	0,194	**0,033**	0,369
10	0,075	**0,009**	0,430
11	**0,007**	**0,000**	0,822
12	0,056	0,134	0,360
13	**0,011**	**0,002**	0,475
14	**0,007**	**0,003**	**0,009**
15	**0,030**	**0,006**	0,872

Table S2. Course of the average internal body temperature (°C) over the test period; pathological values are written in bold

Day	Group							
	Negative Control	PG2T	PLM U	PLM V	PLM W	PLM X	PLM Y	PLM Z
-5	38,9	**39,6**	nb	nb	39,5	nb	nb	nb
-4	39,1	**39,7**	39,5	**39,9**	39,5	**40,0**	39,3	39,6
-3	38,7	39,1	39,2	39,3	39,2	39,0	39,1	39,1
-2	39,1	39,1	39,5	39,3	39,3	39,0	39,0	38,9
-1	38,9	39,2	39,0	38,8	39,1	38,7	39,2	39,0
0	38,8	39,0	39,2	38,9	39,2	38,8	39,1	38,9
0, 2 h p.i.	38,7	**39,6**	**39,9**	39,1	**39,6**	38,9	39,5	39,1
0, 4 h p.i.	39,0	39,5	**39,9**	**39,6**	**39,6**	39,0	**39,7**	**40,2**
0, 8 h p.i.	38,9	**39,6**	**39,8**	**39,8**	39,5	38,8	39,4	**39,9**
0, 12 h p.i.	38,9	**39,6**	**39,8**	**39,8**	39,5	38,8	39,1	**39,7**
1	38,9	38,9	39,2	38,9	38,7	38,8	39,1	38,8
2	38,6	39,4	38,8	39,1	39,1	**40,0**	39,1	38,8
3	38,8	**39,7**	39,0	39,0	39,1	**39,8**	39,5	39,4
4	38,6	**39,6**	39,3	39,2	39,1	**39,9**	39,3	39,4
5	38,6	**39,6**	**40,0**	39,3	39,3	**39,6**	39,5	39,4
6	38,8	39,3	39,3	39,3	39,4	39,0	39,2	38,9
7	38,7	39,2	39,3	39,2	39,1	38,8	38,8	38,9
8	38,6	39,1	39,0	39,1	39,2	38,7	38,9	38,8
9	38,6	39,2	39,0	38,6	39,1	38,6	38,9	39,0
10	38,8	39,2	39,1	39,1	39,4	38,9	38,8	38,9
11	38,7	39,1	38,6	39,1	39,0	39,3	38,7	38,7
12	38,6	38,7	38,8	39,1	39,0	38,6	38,8	38,7
13	38,6	38,9	38,9	38,9	39,4	38,7	38,8	38,9
14	38,7	38,9	38,9	38,7	39,0	38,7	39,0	38,8
15	38,6	39,0	39,0	38,7	39,2	38,9	38,9	38,9
16	nb	38,9	39,0	38,9	39,0	38,9	39,0	39,0

17	nb	38,8	nb	nb	39,2	39,0	nb	39,0
18	nb	39,0	nb	nb	nb	nb	nb	nb

Table S3. Calculation of significant differences in the milk yield from the infected right udder halves between the groups (significant differences in bold). Significance was calculated using the post hoc test. An error probability of $p < 0.05$ was assumed to be significant.

Milk Yield (Right Udder halves)			
Day	Negative Control/ PG2T	Negative Control/ PLM	PG2T/ PLM
-4	1,000	1,000	1,000
-3	1,000	1,000	1,000
-2	1,000	1,000	1,000
-1	0,402	0,448	1,000
0	0,101	0,093	1,000
1	0,181	**0,021**	1,000
2	**0,001**	**0,000**	1,000
3	**0,002**	**0,000**	1,000
4	**0,000**	**0,000**	1,000
5	**0,001**	**0,000**	1,000
6	**0,000**	**0,000**	1,000
7	**0,010**	**0,001**	1,000
8	**0,001**	**0,000**	1,000
9	**0,000**	**0,000**	1,000
10	**0,001**	**0,000**	1,000
11	**0,007**	**0,001**	1,000
12	**0,012**	**0,003**	1,000
13	**0,010**	**0,002**	1,000
14	**0,009**	**0,001**	1,000
15	**0,003**	**0,000**	1,000

Table S4. Hyperplasia of the right (A) and left (B) udder lymph node.

(A)

Group			Frequency
**Negativ**	Valid	.00	6
**PG2**	Valid	1.00	2
		2.00	3
		2.50	1
		Total	6
**PLM_U**	Valid	2.00	3
**PLM_V**	Valid	1.50	1
		2.00	2
		Total	3
**PLM_W**	Valid	2.00	1
		3.00	2
		Total	3
**PLM_X**	Valid	1.00	1
		2.00	2
		Total	3
**PLM_Y**	Valid	2.00	2
		3.00	1
		Total	3
**PLM_Z**	Valid	.00	1
		1.00	1
		2.00	1
		Total	3

(B)

Group			Frequency
**Negativ**	Valid	.00	6
**PG2**	Valid	.00	6
**PLM_U**	Valid	.00	3
**PLM_V**	Valid	1.00	1
		1.50	1
		2.00	1
		Total	3
**PLM_W**	Valid	.00	3
**PLM_X**	Valid	.00	1
		2.00	2
		Total	3
**PLM_Y**	Valid	.00	3
**PLM_Z**	Valid	.00	2
		1.00	1
		Total	3
